# Typologies of Loneliness, Isolation and Living Alone Are Associated with Psychological Well-Being among Older Adults in Taipei: A Cross-Sectional Study

**DOI:** 10.3390/ijerph17249181

**Published:** 2020-12-08

**Authors:** Hui-Chuan Hsu

**Affiliations:** School of Public Health, Research Center of Health Equity, College of Public Health, Taipei Medical University, Taipei 11031, Taiwan; gingerhsu@tmu.edu.tw

**Keywords:** loneliness, isolation, living arrangement, depressive symptoms, life satisfaction, psychological well-being, older adults

## Abstract

Background: Loneliness, isolation, and living alone are emerging as critical issues in older people’s health and well-being, but the effects are not consistent. The purpose of this study was to examine the clustering of loneliness, isolation, and living alone, the risk factors and the associations with psychological well-being. Methods: The data were collected from the 2019 Taipei City Senior Citizen Condition Survey by face-to-face interviews and included a community-based sample (*n* = 3553). Loneliness, isolation, and living arrangement were analyzed by cluster analysis to define Loneliness-Isolation-Living-Alone clusters. Multinomial logistic regression was used to examine the factors related to Loneliness-Isolation-Living-Alone clusters, and linear regression was used to examine association of clusters with psychological well-being. Results: Five clusters of older adults were identified and named as follows: *Not Lonely-Connected-Others* (53.4%), *Not Lonely-Isolated-Others* (26.6%), *Not Lonely-Alone* (5.0%), *Lonely-Connected* (8.1%), and *Lonely-Isolated-Others* (6.9%). Demographics, financial satisfaction, physical function, family relationship, and social participation were related to the Loneliness-Isolation-Living-Alone clusters. Compared with the *Not Lonely-Connected-Others* cluster, the *Lonely-Connected* cluster and *Lonely-Isolated-Others* cluster had higher depressive symptoms and lower life satisfaction, and the *Not Lonely-Isolated-Others* cluster reported lower life satisfaction; the *Not Lonely-Alone* cluster was not different. Discussion: Loneliness and isolation are negatively associated with psychological well-being, and living arrangement is not the determinant to loneliness or isolation. Older adults are suggested to strengthen their informal social support, and the government may encourage social care and create an age friendly environment to reduce loneliness and isolation.

## 1. Introduction

Loneliness, isolation, and living alone (LIL) are emerging as critical issues in older people’s health and well-being. Existing literature has examined the relationship of LIL with physical health, mental health and well-being [1,2,3]. Loneliness is a subjective feeling. It is often defined as a negative feeling due to a lack of intimate social relationships, especially the gap between what one wants and what one truly has regarding social relationships [4]. Isolation is defined as the psychological or physical distance between one individual and other people, or the gap in the social network. Isolation can be represented as lacking social connectedness [5], lacking meaningful, engaging, and quality social relationships in social contacts [6], or having subjective feelings of lacking belongingness or intimacy [6,7]. Living alone is one form of living arrangement that requires the social function or resources from the social network. However, the effects of LIL are often intertwined, but the effects on health are not consistent. The purpose of this study was to identify the clusters of loneliness, isolation, and living alone and the associations of LIL patterns with psychological well-being.

The effects of LIL on health or well-being can be explained by stress [8]. Due to life events, stress influences an individual’s hypothalamic-pituitary-adrenal axis in the endocrine and nervous systems, reduces immunity, and changes behavioral and cognitive status and is adjusted by an individual’s resilience, coping ability and social support. Loneliness or isolation may be viewed as a long-term source of stress. When such stress effects last a long time, they may cause significant effects on health.

Past studies have explored the relationship of loneliness, isolation and living alone on the health and well-being of older people. LIL may cause depression, cardiovascular disease, reduced quality of life, low self-rated health, anxiety, reduced cognitive or physical function, frailty, insomnia, mortality, suicide, and work disability in older adults [1,3,9,10,11,12,13,14,15,16,17,18,19,20,21]. Such negative effects may be worse for individuals with lower education, lower income, and disability [16].

However, the effects of LIL on physical and psychological health are not consistent. Regarding cognitive function, Grande et al. found the effects of living alone related to mild cognitive impairment [22], but other studies from Evans et al. and Kuiper et al. indicated that the effect of living alone is not significant [13,23]. Yang et al. (2020) defines social isolation by living alone, family contact and social engagement, and social isolation showed direct effect on reduced cognitive functioning and through mediating by loneliness [24]. A longitudinal study shows that loneliness may affect cognitive function and increase the risk of dementia, but the isolation effects on cognitive function are not stable, and living alone is not significantly related to dementia [25]. Regarding emotional health, loneliness is found to be related to depressive symptoms but irrelevant to isolation [26]. A longitudinal study indicates that living alone is related to emotional loneliness, but living alone is not related to social isolation [27]. Another study found that living alone is related to depressive symptoms, but such an association is no longer significant when loneliness is controlled [28]. It seems that the effects of loneliness, isolation, and living alone on health and well-being may be different; they simply often happen together. It is necessary to examine their joint effects. Smith and Victor [29] used latent class analysis to examine the combinations of LIL and associations with physical and psychological health. They identified six groups of older people: no loneliness or isolation, moderate loneliness, living alone, moderate isolation, moderate loneliness and living alone, and high loneliness and moderate isolation. These groups were associated differently with physical function, self-rated health, chronic diseases, and depressive symptoms.

The factors related to LIL can be viewed by individual level (demographics, health and psychological characteristics), interpersonal level (family relationship and marital status), and society level (culture, social environment and age friendliness). The factors include age, sex, race, education, socioeconomic status, marital status or widowhood, working status, having children or not, social relationship with friends and neighbors, social anxiety, Internet use, self-rated health, disability, resilience, previous depression, and built environment [10,21,29,30,31,32,33,34,35,36,37,38,39,40,41,42,43]. In addition, social participation [44], activity engagement [45], bonding social capital [46], and religious belief or spirituality [47,48] may also be protective factors. Through participating in social activities, not only the social connection and social support are increased, but also the sense of achievement and feeling of belonging can be improved. In addition, the social environment may be related to loneliness or the effect of loneliness on well-being, such as public space walkability and accessibility [49].

Family relationships are especially important for a family-centered culture such as the traditional culture in Taiwan. According to tradition, Taiwanese people expect to live with family members, to be financially supported by their adult children in old age as a performance of filial piety. In the tradition, older people also did not expect to work or participate in social groups in the old age. However, due to modernization and change of social values, the rate of living with children or grandchildren has reduced in recent decades. Older people who live alone or only live with their spouse are more likely to be male, and also provide and received less support from those who live with family [50]. Loneliness and social isolation are not just lack of companionship, but also represent a state of mind: the gap between desire and reality in the context of culture [51]. In the family-centered Taiwanese culture, the feeling of loneliness may be affected by the gap of expectation and reality of family support. At the same time, the active aging concept is raised and social participation is promoted and gradually accepted among older people in Taiwan society. Age-friendly environments are advocated by World Health Organization [52] and applied in local governments in Taiwan. Age friendliness is found to be related to older people’s well-being [53] and physical domain of health-related quality of life [54]. Therefore, not only may LIL combined with an individual’s characteristics affect well-being, age friendliness should be considered as a factor to mental well-being. 

Despite the existing findings about the relationship of LIL with health and well-being, only a few studies have tried to identify the intertwined relationship of LIL [29], and the inconsistent effects of LIL on health and well-being need further investigation. Therefore, the purpose of this study was to identify the possible combinations of loneliness, isolation, and living alone among community-based older adults and to examine the effects of LIL combinations on psychological well-being in the case of Taipei city.

## 2. Materials and Methods

### 2.1. Data and Sample

The data used for the present study were secondary data collected by face-to-face interviews for the “2019 Taipei City Senior Citizen Condition Survey”. The sample was drawn from citizens whose household was registered in Taipei city. The community-based sample was drawn by stratified sampling (by age and sex stratification in the 12 districts of Taipei city) and systematic sampling (by address and age), and the institutional sample was drawn by stratified random sampling by different types of institutions (nursing homes and long-term care facilities). In total, the completed sample was composed of 3853 persons (3553 persons in the community and 300 persons in institutions). In this current study, only data from the community-based sample were included in the analysis (*n* = 3553). The data was deidentified when released. The study obtained the approval of the Taipei Medical University Joint Institutional Review Board (No. N202010050).

### 2.2. Measures

#### 2.2.1. The Main Independent Variables: LIL

Loneliness was measured by one item from the CES-D scale (do you feel lonely?) [55], and the score was defined as yes (sometimes or often, one to seven days a week)/no (never), as the application of previous research [12,24]. Living alone was defined by the living arrangement, coded as living alone, and living with others in the household. Isolation was defined by the frequency of informal social contact, by asking the respondents, “How often do you have social interaction with friends, relatives, or colleagues, including meeting in person, by telephone, or internet in the past three months? (Interaction because of work does not count)”. The social contact frequency was measured as never, less than once a month, once a month, two to three times a month, once a week, two to six times a week, or every day. If an individual had social contact less than once per week, he or she was described as isolated.

#### 2.2.2. The Dependent Variables

Psychological well-being included two variables: depressive symptoms and life satisfaction. Life satisfaction was scored from 1 to 5 to represent very unsatisfactory to very satisfactory. The other dependent variable was depressive symptoms, which were measured by the CES-D 11-item scale [55] except for the loneliness item. In this survey, each item was scored 1 to 3, so the total 10 items scored from 10 to 30.

#### 2.2.3. Other Covariates

Demographics included age (age 60–64, 65–74, and 75+), sex, education (illiterate, informal education or elementary school, primary high school, senior high school, college/university or above), financial satisfaction (scored 1–5, indicating very unsatisfied to very satisfied), and working status (yes/no).

Health condition variables included self-rated health (score 1–5, indicating worst to excellent), chronic disease numbers (endocrine disease, neuropsychiatry disease, cardiovascular disease, respiratory system disease, digestive system disease, skin disease, skeletal or joint disease, urinary and reproductive system disease, injuries, and other chronic disease), cognitive function, activities of daily living (ADLs), and instrumental activities of daily living (IADLs). Cognitive function was measured by Short Portable Mental State Questionnaire (SPMSQ) [56], scored 0 to 10. ADLs included six items: dressing, transferring, walking indoors, using a toilet, taking a bath, and eating. Each item was measured for performance difficulty and scored 0 to 3. The total score ranged from 0 to 18; a high score indicated more difficulties. IADLs included nine items: shopping for groceries, preparing food, using a telephone, going out by transportation, managing money, doing light housework, doing heavy housework, taking medicine, and doing laundry. Each item was measured by the difficulty, scored 0 to 3; the total score of the difficulties was from 0 to 27.

Social support and social participation included marital status (having a spouse or not), children (yes/no), participation in volunteering (yes/no), political participation (yes/no), religious activity (yes/no), other social group participation (yes/no), and satisfaction with family relationships (recoded as unsatisfied, no opinion or no family, and satisfied).

Age friendliness was measured by three items: how much do you think the public respects older people, does your neighborhood/community hold the events considering older people’s needs, and do you think the products/services provided by society consider older people’s needs? Total scores ranged from 5 to 15; a higher score indicated more age friendliness. 

### 2.3. Analysis

The data analysis was conducted by IBM SPSS 22.0 (IBM, SPSS Inc., Chicago, IL, USA). Descriptive analysis and bivariate tests were conducted to examine the relationships among the variables. Loneliness, isolation, and living alone were analyzed by cluster analysis. The two-step cluster analysis in SPSS procedure was conducted to suggest the best cluster numbers, and then k-means cluster analysis was used to define the Loneliness-Isolation-Living Alone (LIL) clusters. Multinomial logistic regression was used to examine the factors related to LIL clusters. Linear regression was used to examine the relationship of the LIL cluster and other factors with depressive symptoms and life satisfaction.

## 3. Results

The sample characteristics are shown in Table 1. The participants who felt lonely represented 15.1% of the sample; 37.9% were isolated (having social contact less than once per week); and 6.4% were living alone. The bivariate analysis of loneliness, isolation, and living arrangement with the characteristics of the participants are shown in the Supplementary Table S1. 

By the two-step cluster analysis, seven LIL clusters were suggested as presented in Table 2: Not Lonely, Connected, and with Others (53.4%); Not Lonely, Isolated, and with Others (26.6%); Not Lonely, Connected, and Alone (3.5%); Lonely, Connected, and with Others (7.1%); Lonely, Isolated, and with Others (6.9%); Lonely, Connected, and Alone (1.0%); and Not Lonely, Isolated, and Alone (1.4%). However, the sample size of the cluster 3, 6, and 7 was small, that was not suitable for further analysis. Thus, some of the small clusters were combined together, and in total five clusters were defined as presented in Table 3: *Not Lonely*, *Connected*, *and with Others (NLCO)*, 53.4%; *Not Lonely*, *Isolated*, *and with Others (NLIO)*, 26.6%; *Not Lonely and Alone (NLA)*, 5.0%; *Lonely and Connected (LC)*, 8.1%; and *Lonely*, *Isolated*, *and living with Others (LIO)*, 6.9%. The bivariate analysis of LIL clusters with the characteristics of the participants are shown in the Supplementary Table S2. 

Table 4 shows the characteristics of the LIL clusters by the multinomial logistic regression analysis. Compared with the reference group the *Not Lonely-Connected-Others (NLCO)* cluster, the older adults who were in the *(Not Lonely-Isolated-Others (NLIO)* cluster were more likely to be lower educated (OR = 0.905, *p* < 0.05), had worse self-rated health (OR = 0.826, *p* < 0.01), had worse cognitive function (OR = 0.866, *p* < 0.01), had lower financial satisfaction (OR = 0.707, *p* < 0.001), feeling less age friendliness (OR = 0.958, *p* < 0.05), be male (OR = 0.825, *p* < 0.01), had children (OR = 1.691, *p* < 0.05), not working (OR = 0.704, *p* <0.01), and did not participate in other social groups (OR = 0.703, *p* < 0.01). The older adults in the *Not Lonely-Alone (NLA)* cluster were more likely to be higher educated (OR = 1.330, *p* < 0.01), had more chronic diseases (OR = 1.433, *p* < 0.05), did not have a spouse (OR = 0.002, *p* < 0.001), and reported lower family satisfaction or had no family (OR = 0.180, *p* < 0.001). The participants in the *Lonely-Connected (LC)* cluster were more likely to be higher educated (OR = 1.167, *p* < 0.05), had worse self-rated health (OR = 0.701, *p* < 0.001), had more IADL difficulties (OR = 1.095, *p* < 0.01), had more chronic diseases (OR = 1.327, *p* < 0.01), had a worse financial satisfaction (OR = 0.621, *p* < 0.001), be female (OR = 1.622, *p* < 0.01), did not have a spouse (OR = 0.414, *p* < 0.001), participated in other social groups (OR = 2.699, *p* < 0.001), and reported lower family relationship or did not have family (OR = 0.342, *p* < 0.001). The participants in the *Lonely-Isolated-Others (LIO)* cluster were more likely to have worse self-rated health (OR = 0.533, *p* < 0.001), had more IADL difficulties (OR = 1.094, *p* < 0.05), had a worse financial satisfaction (OR = 0.464, *p* < 0.001), were older than 75 years old (OR = 2.006, *p* < 0.05), did not have a spouse (OR = 0.492, *p* < 0.001), and reported lower family relationship or had no family (OR = 0.165, *p* < 0.001). 

Table 5 shows the relationships of the LIL clusters and other factors with depressive symptoms and life satisfaction. Compared with those in the *NLCO* cluster, the older adults in the LC cluster (β = 2.378, *p* < 0.001) and in the LIO cluster (β = 2.674 *p* < 0.001) had higher depressive symptoms. In addition, who were younger, had children, not satisfied with family relationship or not family, lower financial satisfaction, worse self-rated health, more chronic diseases, more IADL difficulties, not participated in social groups and feeling lower age friendliness, had more depressive symptoms. The explained variance (R square) was 0.412.

Regarding the model of life satisfaction, two models were analyzed. In Model (a), the older adults who were in the NLIO cluster (β = −0.058, *p* < 0.05), the LC cluster (β = −0.156, *p* < 0.01) and in the LIO cluster (β = −0.202, *p* < 0.001) had lower life satisfaction than the NLCO group. However, when depressive symptom was controlled in Model (b), the NLIO, LC and LIO clusters were not significant anymore. In addition, the older adults who were older than 75 years old, female, higher educated, satisfied with family relationship, having higher financial satisfaction, feeling more age friendliness, and having fewer depressive symptoms, reported higher life satisfaction. The explained variance (R square) was 0.369. 

## 4. Discussion

This study examined the intertwining relationship of loneliness, isolation, and living alone (LIL) to identify clusters among older adults and related factors, as well as the associations of the LIL clusters with depressive symptoms and life satisfaction in the case of Taipei city. Five LIL clusters were identified: *Not Lonely-Isolated-Others* (53.4%), *Not Lonely-Isolated-Others* (26.6%), *Not Lonely-Alone* (5.0%), *Lonely-Connected* (8.1%), and *Lonely-Isolated-Others* (6.9%). The factors related to LIL clusters included age, sex, education, physical function (IADLs), chronic disease numbers, marital status, having children, working status, family relationship satisfaction, financial satisfaction, and social participation. Compared with those in the *Not Lonely-Connected-Others* cluster, the older adults in the *Lonely-Connected* cluster and the *Lonely-Isolated-Others* cluster had more depressive symptoms and lower life satisfaction; the *Not Lonely-Isolated-Others* cluster did not have more depressive symptoms but reported lower life satisfaction; and the *Not Lonely-Alone* cluster was not significantly different.

### 4.1. Typology of LIL Cluster

The five LIL (*NLCO*, *NLIO*, *NLA*, *LC*, *LIO*) clusters show the mutual relationships of loneliness, isolation, and living alone. The typology is similar to what was found in the study of Smith and Victor [29], which identified six groups by latent class analysis, which indicated the different levels of loneliness and isolation. When controlling for other variables, living alone is not necessarily associated with feeling more isolated or lonely. This means that even if older adults live alone, they can still be socially active and connected. In other words, even though older people live with others, physical strain, poor family relationships, ignorance, or lack of social participation may make them isolated. The social welfare policy in Taiwan focuses on those living alone, but lonely or isolated older people are not noticed under the current system. The individual’s living arrangement in itself may not be the most useful indicator for developing social care for older people.

### 4.2. Factors Related to LIL Clusters

The association of physical function with LIL clusters was found, but no connection was found with cognitive function. The factors related to the five LIL clusters included demographics, IADL difficulties, family relationships, marital status, and social participation. The older adults in the LC cluster and the LIO cluster had more IADL difficulties. The LIO cluster was older than other clusters. It is possible that frailty prevents older adults in the LIO cluster from interacting socially and participating in social life and thus makes them more isolated. Previous research indicates that ADL difficulties are related to loneliness [10,49]. This means that basic physical function limitations may not be the barriers to social connectedness or social participation in this sample, but IADLs need advanced physical function, which may be related to the activities of social interaction or social participation. Cognitive function was not significantly related to the LIL clusters except for the NLIO cluster. The lower cognitive function may be a challenge for the older adults to interact with other people, and thus they were more isolated. There may be two reasons that other clusters were not significantly related to LIL clusters. One reason is that the survey only investigated those who could respond and communicate. Another reason is that the measurement of cognitive function by the SPMSQ was not sensitive; most of the participants showed a high SPMSQ score, and there was no variation. Thus, cognitive function was not used as the dependent variable but was used as a controlling variable in the models. The older participants in the NLA cluster were not significantly different from the NLCO group in physical function or cognitive function. That means the NLA cluster adults are able to live independently and live alone. 

Social network and family support are important factors in LIL clusters. Those who did not have spouses, had no family or were not satisfied with family relationships were more likely to be in the NLA, LC, and LIO clusters, i.e., living alone or feeling lonely. The results were consistent with previous studies [9,10,29,30,31]. The reason to live alone for the NLA cluster is probably because they did not have family or did not expect to live with family. Family relationship satisfaction was significant not only for those who did not have family, but also significant for those who had family or were living with family, especially the two lonely groups (LC and LIO). It is possible that the older adults have unfulfilled expectations on their family, and thus the feeling of loneliness was stronger. 

The NLIO cluster needs further attention. The NLIO cluster was not significantly related to marital status; however, it was related to having children and working. The participants in the NLIO cluster also were more likely to be male, less likely to participate in social groups, and reported lower age friendliness. The NLIO cluster is similar to the isolated group in the study of Smith and Victor [29]. It is possible that the members of the NLIO cluster may live with family or have contact in their occupation, but they did not have much informal social interaction with family and friends, especially for older men. The lifestyle for NLIO cluster may be less socialized. 

Participating in volunteering, religious activity were not different across cluster. Participating in other social groups were higher in the LC cluster (OR = 2.685) and lower in the NLIO cluster (OR = 0.703). Participating in social groups is one kind of social contact, which prevents older people from getting isolated by connecting with other people. Gale et al. [15] found that those who are socially isolated (no social contact and no social group participation) were more likely to be lonely. However, the effects of social group participation on its own on loneliness are not confirmed. One of the possible reasons for the association of loneliness and social group participation is that social group participation is a shallow interaction without engagement for the participants. Another explanation is that older adults participate in these social groups because they feel lonely and would like to reduce their loneliness by participation in a social group. Because this is a cross-sectional study, the causal relationship cannot be confirmed. 

The *NLIO*, *LC*, and *LIO* older adults had lower financial satisfaction. The three clusters were either isolated or lonely. Chung et al. [57] define financial social exclusion as one type of social exclusion: financial strain may cause barriers in maintaining social relationships, and life satisfaction can be lower. 

### 4.3. LIL Clusters and Psychological Well-Being

The two lonely cluster of older adults, LC and LIO, had more depressive symptoms and lower life satisfaction, which is consistent with previous studies [11,29,30,33,36]. When the depressive symptom variable was added, the effects of LIL clusters were not significant. That implies depressive symptoms are the mediators of loneliness to life satisfaction. The NLIO group did not report higher depressive symptoms, but they still showed significantly lower life satisfaction even when the depressive symptom was controlled for. The older adults in the NLIO cluster were isolated, had less family interaction and were more likely to be males. One explanation is that the NLIO cluster older adults did not recognize their loneliness [29]. The other explanation is that the social relationship was not positive for these older adults or there were unmet needs in their social relationship [39], or some of the older adults had social anxiety in social interactions [40]. Thus, social isolation did not influence their mental health (depressive symptoms). However, both isolated clusters (NLIO, LIO) had lower life satisfaction. Social connectedness is one of the indicators used to measure active aging [58], and social connectedness provides social capital, which is beneficial for resources and reciprocity [46]. Although not all social relationships are positive, having a moderate social network and appropriate social support may still be positive for well-being. The reasons for being isolated should be explored in qualitative research in the future.

Depressive symptoms and life satisfaction for the NLA older adults were not different from the NLCO cluster. Living alone is not necessarily equal to a lack of social connectedness and social participation [27], and loneliness but not living alone is associated with depressive symptoms [28]. It is also possible that the older adults in the NLA cluster may have intact physical function and cognitive function to live alone, and thus their psychological well-being was not affected by living arrangement. The NLA cluster also had higher education, indicating a better socioeconomic status and better chance to well-being. Although the older adults in the NLA cluster live alone or did not have family, if the living-alone adults have independent health and live in a meaningful, connected, and active lifestyle, their mental health and well-being can still be fine. 

Lower financial satisfaction was related to more depressive symptoms and lower life satisfaction. A study from Europe indicates that individuals have more worries of retirement income if their country is projecting more population aging and more income inequality [59]. It seems that financial security is related to psychological well-being, especially when the older adults do not have confidence in social support, such as the *Lonely* or the *Isolated* older adults in this study. Old age policy should provide a good basis for pension and security for older people and trust in the government so that older people (especially isolated or lonely people) do not need to additionally worry about their financial security. In addition, age friendliness was related to lower depressive symptoms and higher life satisfaction, similar to previous research [53,54]. If the community or society is age-friendly, the older people would feel more comfortable and more willing in social interaction and social participation, which would thus reduce isolation and loneliness. 

### 4.4. Limitations

There are some limitations in this study. First, the data were cross-sectional data. The causal relationships of LIL with other variables cannot be confirmed. Second, the sample was drawn from older adults living in Taipei city. Taipei city is an urban modern city. The infrastructure for an age-friendly environment is better, and the access to the health and community services is much abundant than in rural areas, which reduces the barrier of social connection and participation. However, the percentage of older adults living with children or grandchildren is lower than that of rural areas, and more social interactions with family, neighbors and friends may be more frequently in rural areas. In addition, the traditional culture in Taiwan is family-centered. Older people usually hope to live with family but not live alone. The results may not be generalizable to other areas, populations or cultures. Third, the data were from secondary data. There may be other confounders or related factors that are not available. For example, loneliness was measured by only a single item, just as past research using secondary data [12,24]. The single-item measure cannot describe loneliness in multiple dimensions. Cognitive function measurement may not be sensitive to detecting different levels of cognitive performance. There may be other confounders or related factors that are not available. Fourth, only the individual characteristics were included in the analysis. The social environment factors across the districts were not analyzed. There were district area differences in LIL clusters. The environmental factors may need further investigation.

## 5. Conclusions

The combinations of loneliness, isolation and living alone exhibit different typologies of the social lifestyle of older adults. Older adults who live alone are not necessarily isolated; isolated older adults do not necessarily feel lonely; older adults living with family can still be isolated or feel lonely. The LIL typologies provide information about the social lifestyle of older adults. 

Social contact and social support, especially a good relationship with the family, may be the key to preventing loneliness and isolation. Older adults living with family may still feel lonely, with needs unfulfilled by their family. Older adults who feel lonely should be made aware of ways to prevent the negative impact of depression. An appropriate quantity of social contact and the quality of social relationships are both important. Older adults are suggested to strengthen their informal social support (including support from friends and neighbors in the community) in case the family support is not enough, and the government may encourage social care provided in the community as extra social support. 

The barrier due to difficulty in physical function may prohibit social interaction or participation, and cause loneliness or isolation. An age-friendly environment would help remove such barriers regarding mobility. The financial security issue may also cause loneliness or isolation. Financial security should be addressed by the government, and a security net for older people should be built. In addition, not all social participation can reduce loneliness or isolation. That implies that meaningful engagement or participation is more important than shallow social interactions. 

Finally, the causal relationship of loneliness, isolation and living alone with psychological well-being should be further investigated by better measurement and longitudinal studies, and the nature of isolation and the corresponding solution should be developed by qualitative research in the future.

## Figures and Tables

**Table 1:** Description of the sample.

Variables	*n* (*N* = 3553)	Mean (SD) or %
Age		
Age 60–64	1069	30.1%
Age 65–69	938	26.4%
Age 70–74	557	15.7%
Age 75–79	423	11.9%
Age 80–84	260	7.6%
Age 85+	296	8.3%
Sex		
Female	1941	54.6%
Male	1612	45.4%
Education		
Illiterate	166	4.7%
Informal education or elementary school	1055	29.7%
Primary high school	517	14.6%
Senior high school	938	26.4%
College/University or above	877	24.7%
Marital status		
No spouse	1123	31.6%
Having spouse	2430	68.4%
Children		
No children	207	83.3%
Having children	3346	16.7%
Financial satisfaction	3184	3.48 (0.78)
Working status		
No	2961	83.3%
Yes	592	16.7%
Self-rated health (1–5)	3184	3.55 (0.84)
Cognitive function (1–10)	3184	9.57 (0.98)
Chronic disease number (0–7)	3553	0.97 (0.96)
Activities of daily living (0–18)	3553	0.68 (2.87)
Instrumental activities of daily living (0–27)	3553	1.87 (5.44)
Depressive symptom1 (10–30)	3184	13.25 (2.68)
Life satisfaction (1–5)	3184	3.88 (0.74)
Age friendliness (3–15)	3164	10.45 (2.43)
Volunteering		
No	3397	95.6%
Yes	156	4.4%
Political participation		
No	3534	99.5%
Yes	19	0.5%
Religious activity		
No	3096	87.1%
Yes	457	12.9%
Other social groups		
No	3022	85.1%
Yes	531	14.9%
Family satisfaction		
Unsatisfied	121	3.8%
No family or no opinion	178	5.6%
Satisfied	2885	90.6%
Living arrangement		
Alone	229	6.4%
With others	3324	93.6%
Loneliness		
Not lonely	2704	84.9%
Lonely	480	15.1%
Isolation		
Isolated (social contact < 1 per week)	1345	37.9%
Connected (social contact ≥ 1 per week)	2208	62.1%

Note: *N* = 3553. Missing cases are excluded listwise.

**Table 2:** Loneliness, isolation, and living alone (LIL) combination distribution of the community-based sample—seven clusters (%).

Variables	Cluster 1: (53.4%) ^1^	Cluster 2: (26.6%) ^2^	Cluster 3: (3.5%) ^3^	Cluster 4: (7.1%) ^4^	Cluster 5: (6.9%) ^5^	Cluster 6: (1.0%) ^6^	Cluster 7 (1.4%) ^7^
Loneliness							
Not Lonely	100.0	100.0	100.0	0.0	0.0	0.0	100.0
Lonely	0.0	0.0	0.0	100.0	100.0	100.0	0.0
Isolation							
Isolated	0.0	100.0	0.0	0.0	100.0	0.0	100.0
Connected	100.0	0.0	100.0	100.0	0.0	100.0	0.0
Living arrangement							
Alone	0.0	0.0	100.0	0.0	15.4	100.00	100.0
With Others	100.0	100.0	0.0	100.0	84.6	0.0	0.0

Note: *N* = 3184. The clusters were grouped by two-stage cluster analysis. ^1^ Not Lonely, Connected, and with Others, ^2^ Not Lonely, Isolated, and with Others, ^3^ Not Lonely, Connected, and Alone, ^4^ Lonely, Connected, and with Others, ^5^ Lonely, Isolated, and with Others, ^6^ Lonely, Connected, and Alone, ^7^ Not Lonely, Isolated, and Alone.

**Table 3:** LIL combination distribution of the community-based sample—five clusters (%).

LIL Clusters	*N* (%)
Not Lonely-Connected-Others (NLCO)	1700 (53.4%)
Not Lonely-Isolated-Others (NLIO)	846 (26.6%)
Not Lonely-Alone (NLA)	158 (5.0%)
Lonely-Connected (LC)	259 (8.1%)
Lonely-Isolated-Others (LIO)	221 (6.9%)

Note: *N* = 3184. The clusters were grouped based on Table 2.

**Table 4:** Factors related to LIL combinations by multinomial logistic regression (odds ratios).

Factors	NLIO (Not Lonely, Isolated, with Others)	NLA (Not Lonely and Alone)	LC (Lonely and Connected)	LIO (Lonely, Isolated, with Others)
Education	0.905 *	1.330 **	1.167 *	0.931
Self-rated health	0.826 **	1.053	0.701 ***	0.533 ***
ADL difficulty	0.954	0.780	0.967	1.006
IADL difficulty	1.034	0.952	1.095 **	1.094 **
Chronic disease number	1.110	1.433 **	1.327 **	0.931
Cognitive function	0.866 **	0.930	0.919	0.883
Financial satisfaction	0.707 ***	1.016	0.621 ***	0.464 ***
Age friendliness	0.958 *	0.995	1.012	1.004
Age 65–74	1.062	1.064	0.853	1.123
Age 75+	1.274	1.395	0.851	2.006 **
Sex (female)	0.825 *	0.705	1.622 **	1.112
Marital status (having spouse)	0.929	0.002 ***	0.414 ***	0.492 ***
Children (yes)	1.691 *	0.815	0.957	0.807
Work (yes)	0.704 **	1.067	0.864	0.764
Volunteering (yes)	1.035	2.081	1.089	1.311
Religious activity (yes)	0.794	0.948	0.748	0.859
Other social group participation (yes)	0.703 **	0.822	2.699 ***	1.076
Family satisfaction (satisfied)	0.776	0.180 ***	0.342 ***	0.165 ***
Model fit	−2LL: intercept only = 7587.422; final model = 6363.982, Chi-square = 1223.440, df = 72, *p* < 0.001.			

Note: Analysis by multinomial logistic regression. Intercept is omitted from the table. −2LL: −2 log likelihood. The reference group: LIL (NLCO: *Not Lonely*, *Connected*, *and with Others)*, sex (male), age (age 60–64), marital status (no spouse), children (no), volunteering (no), religious activity (no), other social group participation (no), family satisfaction (unsatisfied or no family). Ordinal or continuous variable: age, education, self-rated health, ADL difficulty, IADL difficulty, chronic disease number, cognitive function, financial satisfaction, age friendliness. * *p* < 0.05, ** *p* < 0.01, *** *p* < 0.001.

**Table 5:** LIL and psychological health and well-being by linear regression.

Variables	Depressive Symptom		Life Satisfaction (a)		Life Satisfaction (b)	
	B	SE	B	SE	B	SE
Constant	17.945	0.507 ***	1.695	0.149 ***	2.711	0.173 ***
**NLIO (not lonely, isolated, with others)**	0.120	0.090	−0.058	0.026 *	−0.051	0.026
**NLA (not lonely and alone)**	0.055	0.189	0.007	0.056	0.010	0.055
**LC (lonely and connected)**	2.378	0.144 ***	−0.156	0.042 ***	−0.021	0.043
**LIO (lonely, isolated, with others)**	2.674	0.161 ***	−0.202	0.047***	−0.051	0.049
Age 65–74	−0.152	0.090	0.036	0.027	0.027	0.026
Age 75+	−0.472	0.118 ***	0.134	0.035 ***	0.107	0.034 **
Sex (male)	−0.068	0.080	−0.052	0.024 *	−0.056	0.023 *
Education	0.019	0.033	0.034	0.010 ***	0.035	0.010 ***
Marital status (having spouse)	−0.060	0.098	0.021	0.029	0.018	0.028
Children (yes)	0.523	0.169 **	−0.020	0.050	0.010	0.049
Family satisfaction (satisfied)	−0.665	0.136 ***	0.808	0.040 ***	0.770	0.039
Work (yes)	−0.116	0.104	<−0.001	0.031	−0.007	0.030 ***
Financial satisfaction	−0.296	0.053 ***	0.302	0.016 ***	0.285	0.015 ***
Self-rated health	−0.906	0.051 ***	0.065	0.015 ***	0.013	0.015
Chronic disease number	0.187	0.046 ***	−0.004	0.014	0.007	0.013
ADL difficulty	0.052	0.044	−0.012	0.013	−0.010	0.013
IADL difficulty	0.123	0.020 ***	−0.003	0.006	0.004	0.006
Cognitive function	−0.036	0.041	−0.006	0.012	−0.008	0.012
Volunteering (yes)	−0.150	0.176	0.014	0.052	0.005	0.051
Religious activity (yes)	−0.143	0.112	−0.015	0.033	−0.023	0.032
Other social group (yes)	−0.524	0.104 ***	0.048	0.031	0.018	0.030
Age friendliness	−0.035	0.016 *	0.013	0.005 **	0.012	0.005 *
Depressive symptoms	---	---	---	---	−0.057	0.005 ***
R square	0.412		0.345		0.369	

Note: Analysis by linear regression. The reference group: LIL (NLCO: the *Not Lonely-Connected-Others* cluster), age (age 60–64), sex (female), marital status (no spouse), children (no), family satisfaction (satisfied), working (no), volunteering (no), religious activity (no), other social group (no). Other variables are ordinal or continuous. * *p* < 0.05, ** *p* < 0.01, *** *p* < 0.001.

## Supplementary Materials

The following are available online at https://www.mdpi.com/1660-4601/17/24/9181/s1, Table S1. Bi-variate analysis of factors related to loneliness, isolation, and living alone, Table S2: Bi-variate analysis of factors related to LIL Clusters.
