# Supplementary material for: Typologies of Loneliness, Isolation and Living Alone Are Associated with Psychological Well-Being among Older Adults in Taipei: A Cross-Sectional Study
Source: Int J Environ Res Public Health. 2020 Dec 8;17(24):9181. doi: 10.3390/ijerph17249181 (PMC7764280; doi:10.3390/ijerph17249181)
Supplement: Supplementary file 1 [file ijerph-17-09181-s001.pdf]

Table S1. Bi-variate analysis of factors related to loneliness, isolation, and living alone.

Variables	Loneliness		Isolation		Living Arrangement	
	Unlonely	Lonely	Isolated	Connected	Living Alone	Living with Others
Age	***		***		*	
Age 60–64	83.7%	12.7%	29.5%	70.5%	4.9%	95.1%
Age 65–74	86.1%	13.9%	35.3%	64.7%	6.8%	93.2%
Age 75+	79.5%	20.5%	50.9%	49.1%	7.6%	92.4%
Sex	***				***	
Female	82.0%	18.0%	36.7%	63.3%	7.8%	92.2%
Male	88.5%	11.5%	39.2%	60.8%	4.8%	95.2%
Education	***		***			
Illiterate	72.3%	27.7%	58.4%	41.6%	6.0%	94.0%
Informal education or elementary school	82.5%	17.5%	44.5%	55.5%	5.9%	94.1%
Primary high school	86.1%	13.9%	36.2%	53.8%	6.2%	93.8%
Senior high school	86.5%	13.5%	32.3%	67.7%	7.2%	92.8%
College/University or above	87.1%	12.9%	27.0%	73.0%	6.5%	93.5%
Marital status	***		***		***	
No spouse	75.8%	24.2%	43.5%	56.5%	20.1%	79.9%
Having spouse	88.7%	11.3%	35.3%	64.7%	0.1%	9.9%
Children	***				***	
No children	75.0%	25.0%	34.8%	65.2%	26.1%	73.9%
Having children	85.5%	14.5%	38.0%	62.0%	5.2%	94.8%
Family satisfaction	***		***		***	
Unsatisfied or no family	59.9%	40.1%	53.5%	46.5%	27.1%	72.9%
Satisfied	87.5%	12.5%	33.0%	67.0%	5.0%	95.0%
Financial satisfaction	3.55 (0.745) ***	3.07 (0.82)	3.26 (0.76) ***	3.50 (0.77)	3.21 (0.97) ***	3.50 (0.76)
Working status	**		***			
No	84.0%	16.0%	40.1%	59.9%	6.5%	93.5%
Yes	89.0%	11.0%	26.7%	73.3%	6.4%	93.6%
Self-rated health	3.62 (0.81) ***	3.14 (0.89)	3.33 (0.81)	3.66 (0.83)	3.48 (0.95)	3.55 (0.83)
Cognitive function	9.62 (0.89) ***	9.31 (1.32)	9.40 (1.18) ***	9.67 (0.83)	9.47 (1.18)	9.58 (0.96
Chronic disease number	0.86 (0.88) ***	1.18 (1.02)	1.17 (0.98) ***	0.85 (0.92)	1.21 (1.04) ***	0.95 (0.95)

Activities of daily living	0.11 (0.94) ***	0.67 (2.29)	1.32 (0.39) ***	0.30 (1.87)	0.16 (0.79) ***	0.72 (2.95)
Instrumental activities of daily living	0.55 (2.39) ***	2.29 (5.00)	3.38 (7.18) ***	0.95 (3.74)	0.88 (2.88) ***	1.94 (5.57)
Volunteering			*		*	
No	84.8%	15.2%	38.2%	61.8%	6.3%	93.7%
Yes	86.8%	13.2%	30.1%	69.9%	10.3%	89.7%
Religious activity			***			
No	84.8%	15.2%	39.1%	69.9%	6.3%	93.7%
Yes	860%	14.0%	29.3%	70.7%	7.7%	92.3%
Other social groups	***		***			
No	86.0%	14.0%	40.3%	59.7%	6.7%	93.3%
Yes	79.3%	20.7%	23.9%	76.1%	4.9%	951%
Age friendliness	10.49 (2.42) *	10.20 (2.44)	10.09 (2.21) ***	10.64 (2.52)	10.02 (2.61)	10.48 (2.41)
Depressive symptom1	12.74 (2.06) ***	16.15 (3.71)	13.9 (12.88) ***	12.90 (2.49)	13.80 (3.32) **	13.21 (2.62)
Life satisfaction	3.95 (0.67) ***	3.46 (0.97)	3.70 (0.82) ***	3.97 (0.68)	3.61 (1.03) ***	3.90 (0.71)

Note: Analysis by Chi-square test or t-test. * $p < 0.05$, ** $p < 0.01$, *** $p < 0.001$.

Table S2. Bi-variate analysis of factors related to LIL Clusters.

Variables	UCO (Unlonely, Connected, living with Others)	UIO (Unlonely, Isolated, living with Others)	UA (Unlonely and living Alone)	LC (Lonely and Connected)	LIO (Lonely, Isolated, and living with Others)	Significance
Age						***
Age 60–64	60.2%	22.7%	4.4%	8.3	4.4%	
Age 65–74	55.1%	26.4%	4.5%	8.1%	5.9%	
Age 75+	40.8%	32.1%	6.6%	8.0%	12.4%	
Sex						***
Female	51.4%	24.7%	5.9%	10.0%	8.0%	
Male	55.8%	28.8%	3.8%	5.8%	5.7%	
Education						***
Illiterate	40.2%	27.7%	4.5%	8.0%	19.6%	
Informal education or elementary school	47.5%	30.9%	4.1%	7.9%	9.6%	
Primary high school	47.9%	33.8%	4.5%	7.3%	6.6%	
Senior high school	56.5%	24.0%	5.9%	8.2%	5.3%	

College/University or above	61.9%	20.0%	5.2%	8.8%	4.1%	
Marital status						***
No spouse	36.9%	22.2%	16.7%	12.5%	11.7%	
Having spouse	60.3%	28.4%	0.0%	6.3%	4.9%	
Children						***
No children	40.8%	13.0%	21.2%	13.6%	11.4%	
Having children	54.2%	27.4%	4.0%	7.8%	6.7%	
Family satisfaction						***
Unsatisfied or no family	24.7%	19.7%	15.4%	15.1%	25.1%	
Satisfied	56.4%	27.3%	39%	7.4%	5.1%	
Financial satisfaction	3.64 (0.75)	3.38 (0.70)	3.42 (0.89)	3.22 (0.81)	2.90 (0.80)	***
Working status						***
No	51.2%	27.9%	4.9%	8.4%	7.6%	
Yes	63.4%	29.5%	5.1%	6.9%	4.1%	
Self-rated health	3.71 (0.80)	3.43 (0.77)	3.62 (0.89)	3.29 (0.91)	2.95 (0.82)	***
Cognitive function	9.70 (0.74)	9.46 (1.09)	9.54 (1.10)	9.47 (1.16)	9.12 (1.47)	***
Chronic disease number	0.76 (0.86)	1.01 (0.86)	1.10 (1.0)	1.17 (1.12)	1.19 (0.90)	***
Activities of daily living	0.07 (0.78)	0.20 (1.26)	0.04 (0.36)	0.44 (2.00	0.95 (2.58)	***
Instrumental activities of daily living	0.37 (1.85)	0.96 (3.25)	0.42 (1.81)	1.64 (4.57)	3.04 (5.37)	***
Volunteering						
No	53.2%	26.7%	4.8%	8.1%	7.0%	
Yes	56.3%	23.2%	7.3%	7.9%	5.3%	
Religious activity						
No	52.6%	27.3%	4.8%	8.1%	7.2%	
Yes	58.4%	21.8%	5.7%	8.5%	5.5%	
Other social groups						***
No	52.5%	28.3%	5.2%	6.7%	7.3%	
Yes	57.9%	17.7%	3.7%	15.4%	5.3%	
Age friendliness	10.68 (2.48)	10.17 (2.220)	10.20 (2.66)	10.43 (2.66)	9.94 (2.14)	***
Depressive symptom1	12.51 (1.95)	13.15 (2.10)	12.89 (2.54)	15.64 (3.77)	16.75 (3.56)	***
Life satisfaction	4.03 (0.62)	3.84 (0.68)	3.76 (0.94)	3.62 (0.89)	3.29 (1.03)	***

Note: Analysis by Chi-square test or one-way ANOVA test. * $p < 0.05$, ** $p < 0.01$, *** $p < 0.001$.
